# Implementation of algorithms in pattern & impression evidence: A responsible and practical roadmap

**DOI:** 10.1016/j.fsisyn.2021.100142

**Published:** 2021-02-18

**Authors:** H. Swofford, C. Champod

**Affiliations:** School of Criminal Justice, Forensic Science Institute, University of Lausanne, Switzerland

**Keywords:** Forensic science, Pattern evidence, Algorithms, Statistics, Models, Automation

## Abstract

Over the years, scientific and legal scholars have called for the implementation of algorithms (e.g., statistical methods) in forensic science to provide an empirical foundation to experts’ subjective conclusions. Despite the proliferation of numerous approaches, the practitioner community has been reluctant to apply them operationally. Reactions have ranged from passive skepticism to outright opposition, often in favor of traditional experience and expertise as a sufficient basis for conclusions. In this paper, we explore *why* practitioners are generally in opposition to algorithmic interventions and *how* their concerns might be overcome. We accomplish this by considering issues concerning human—algorithm interactions in both real world domains and laboratory studies as well as issues concerning the litigation of algorithms in the American legal system. Taking into account those issues, we propose a strategy for approaching the implementation of algorithms, and the different ways algorithms can be implemented, in a *responsible* and *practical* manner.

## 1 Introduction

Over the years, the forensic science community has faced increasing criticism from scientific and legal scholars, challenging the validity and reliability of many forensic examination methods that rely on subjective interpretations by forensic practitioners [[1], [2], [3], [4], [5], [6], [7], [8], [9]]. Of particular concern is the lack of an empirically demonstrable basis to substantiate conclusions from pattern and impression evidence, which has led to calls for reform through the development and integration of tools to evaluate and report the strength of forensic evidence using validated statistical methods [3,[7], [8], [9]]. Some, such as the President’s Council of Advisors on Science and Technology (PCAST), have gone so far as to suggest forensic analyses should be fully objective such that “they can be performed by either an automated system or human examiners exercising little or no judgment” [8]. As illustrated by the PCAST, algorithms and automation are often proposed as a natural solution to the limitations of human judgment. Although concerns over subjective interpretation and lack of statistical evidence span across most pattern and impression evidence domains, the practice of friction ridge examination is often a focal point of debate due to its long-standing history and ubiquitous practice. In the friction ridge discipline in particular, there have been a number of notable efforts by researchers for which algorithms have been introduced to provide quantitative or statistical approaches to the analysis and evaluation of evidence [[10], [11], [12], [13], [14], [15], [16], [17], [18], [19], [20], [21], [22], [23], [24], [25], [26], [27], [28], [29], [30], [31], [32], [33], [34], [35], [36], [37]]. Despite the proliferation of proposed methods, however, the practitioner community has been reluctant to apply them operationally. Reactions toward the intervention of statistical methods, even statistical *concepts*, have ranged from passive observation and skepticism to outright opposition [[38], [39], [40], [41]]. Reasons cited for these reactions are expansive—often resulting in an excuse for the whole-sale rejection of available methods in favor of traditional experience and expertise [41]. Fueled by perceptions that algorithms can only be implemented as an “all or nothing” approach (*either* the human *or* the algorithm), this has led to controversy and opposing viewpoints between many in the scientific and practitioner communities which have ultimately created a stalemate. On the one hand, opponents of algorithmic interventions might point to anecdotal instances in which the algorithms have failed as proof that technologies are inferior and highlight concerns of new challenges that have yet to be understood and fully explored when algorithms are implemented without proper scrutiny, training, oversight, and quality controls [42]. On the other hand, proponents of algorithmic interventions might too easily brush off negative reactions toward algorithms and characterize them as irrational or hyperbolic seeking to maintain traditional practices and preserve autonomous decision making.

What has become clear over the last decade is that calls for algorithms in forensic science are unlikely to subside and challenges to implementation are unlikely to be solved by improvements to technology or the mere proliferation of the tools alone. The problem is much more complicated and requires careful consideration of different issues. First, we need to take a step back and better understand *why* practitioners are hesitant to rely on algorithms and *how* their concerns might be overcome to increase receptivity. This will require us to look outside forensic science in other domains where algorithms have been introduced and consider the issues through the lenses of psychology and behavioral sciences as it relates to human—algorithm interactions. Then, we need to consider the environment in which forensic science operates and to which the algorithms will ultimately be applied—the criminal justice system—and the impact algorithms can have on sensitive decisions impacting life and liberty of citizens. Finally, with these contexts in mind, we need to consider how to mitigate the concerns of forensic practitioners and criminal justice stakeholders and navigate a way forward for the implementation of algorithms into forensic science in a *responsible* and *practical* manner. In most circumstances, hastily jumping from no algorithmic influence, which represents the current state of forensic science today, to complete automation, as envisioned by PCAST, without a clear roadmap and consideration of the complex and dynamic issues at play is both irresponsible and impractical. To this end, in this paper we will (i) outline the foundations that need to be in place from a quality assurance perspective before algorithms should be implemented, such as education, training, protocols, validation, verification, competency, and on-going monitoring schemes, and (ii) propose a taxonomy of six different levels of algorithm implementation ranging from Level 0 (no algorithm influence) to Level 5 (complete algorithm influence) describing various ways in which algorithms can be implemented. In levels 0 through 2, the human serves as the predominant basis for the evaluation and conclusion with increasing influence of algorithms as a supplemental factor for quality control (used *after* the expert opinion has been formed). In Levels 3 through 5, algorithms serve as the predominant basis for the evaluation and conclusion with decreasing influence from the human. We note that this taxonomy is distinct from levels of technology readiness often used to describe the maturity of technology for operational deployment (e.g., see Ref. [43]); the levels outlined in our proposed taxonomy applies to algorithms that have been validated and are ready for operational deployment. This taxonomy, therefore, not only provides a common foundation to communicate what it means for an algorithm to be implemented and the degree to which algorithms influence the overall outcome of the evaluation at each level, but it also provides a framework for forensic science disciplines to implement algorithms in a deliberate and progressive way that is considerate of the implications algorithms will have on traditional examination practices as well as the criminal justice system and its stakeholders.

In the discussion that follows, we take an agnostic viewpoint of any specific method and instead frame the issue on the topic of integrating algorithms (in general) into domains that are largely driven by human judgment. For these purposes, the term “algorithm” is used to broadly describe any evidence-based prediction method, such as statistical models, decision rules, and other mechanical processes used for forecasting, predictions, statistical evaluations and decision making. We approach this discussion in five parts. In Part I, we start by taking a retrospective look at the challenges faced with the initial introduction of algorithms into the scheme of clinical decision making, with particular emphasis on medical practitioners—a domain we consider a reasonable proxy for exploring issues related to human—algorithm interaction in forensic science. In Part II, we discuss issues concerning human—algorithm interactions more generally and summarize key research findings from psychology and behavioral sciences regarding the tendency for people to rely on algorithms and factors that are believed to increase or decrease those tendencies. In Part III, we consider the generalizability of the research findings in the context of two real-world domains that have traditionally relied on human judgment based on intuition and experience and where human—algorithm interactions have naturally begun to take shape: medicine and autonomous vehicles. In Part IV, we discuss specific challenges related to the introduction of algorithms into the American legal system. Finally in Part V, we build on the discussion from prior sections and propose a path forward for the integration of algorithms into forensic practice that is believed to increase the likelihood for adoption across all stakeholders and lead to an overall stronger foundation and improvement to the quality and consistency of forensic science. We note that in the various parts throughout this review, we provide several (sometimes lengthy) quotations from key papers. This is done to ensure we do not distort the authors’ original positions or views related to specific issues discussed and to enable readers to discern the similarities and applicability to the current state of forensic science.

### 1.1 Part I: the introduction of algorithms in clinical decision making

Leading up to the 1950’s, there were growing debates in the scientific and medical communities on the superiority of predictions made on the basis of clinical judgment (e.g. subjective, experience-based) vs. statistical methods (e.g. algorithmic, actuarial). Theoretical arguments divided the two communities (often down parting lines of clinicians vs. statisticians) and proponents for each paradigm asserted the answers were ‘obvious’ [44]. In 1954, Paul Meehl, an clinical psychologist, explored this issue and published a landmark book entitled *Clinical versus Statistical Prediction: A Theoretical Analysis and Review of the Evidence* in which he considers the theoretical arguments from both sides and reviews results of twenty different forecasting studies across diverse domains, including academic performance and parole violations. This was the first known empirical study comparing the relative performance of clinical judgment versus statistical methods (e.g. linear models) for prediction tasks. Meehl finds that predictions based on statistical methods consistently outperformed those based on the judgment from skilled human counterparts [44]. Shortly after publication, Meehl’s findings were met with skepticism by other clinical experts. In his book *Thinking Fast and Slow*, Kahneman recounts “[f]rom the very outset, clinical psychologists responded to Meehl’s ideas with hostility and disbelief.” [45]. In the years that followed, Meehl’s work stimulated a proliferation of research on the topic of clinical versus statistical methods for prediction tasks. Study after study, researchers repeatedly reported the superiority of algorithms versus humans [46,47]. Grove et al. (2000) provides a meta-analysis on 136 studies over the last four decades and finds overwhelming evidence demonstrating statistical methods performing on par with or better than human judgment across a variety of domains, including medical diagnoses, mental health, psychology, academic success, parole violations, business operations, personnel decisions, and more [47]. The authors summarize their findings as “[o]n average, mechanical-prediction techniques were about 10% more accurate than clinical predictions” and “[s]uperiority for mechanical-prediction techniques was consistent, regardless of the judgment task, type of judges, judges’ amounts of experience, or the types of data being combined.” [47]. Thirty years after his original publication, Meehl published a commentary regarding his original 1954 publication in which he recounts the reactions of his fellow clinicians suggesting he was “fomenting a needless controversy” and offers his views following three decades of reflection [48]. In his commentary, Meehl notes [48]:

There is no controversy in social science that shows such a large body of qualitatively diverse studies coming out so uniformly in the same direction as this one. When you are pushing 90 investigations, predicting everything from the outcome of football games to the diagnosis of liver disease and when you can hardly come up with half dozen studies showing even a weak tendency in favor of the clinician, it is time to draw a practical conclusion, whatever theoretical differences may still be disputed. Why, then, is such a strongly and clearly supported empirical generalization not applied in practice, particularly because there are no plausible theoretical reasons to have expected otherwise in the first place?

With mounting evidence demonstrating the superiority of algorithms over subjective judgment, it would seem logical for people to welcome algorithms for these tasks with open-arms. However, they often *don’t*. Some of the anecdotal reasons cited for the reluctance to rely on algorithms include the presumed inability of algorithms to incorporate qualitative data [46], the notion that algorithms cannot properly consider individual circumstances [46], the notion that algorithms are dehumanizing [46,49], the inability of algorithms to learn [49], concerns about the ethicality of relying on algorithms to make important decisions [49], the desire for perfection [49,50], and the presumed ability of humans to improve through experience [50]. In their article, Grove & Meehl [46] challenge common objections from clinicians regarding the use of algorithms in practice and suggest “some of the sociopsychological factors that may help to explain this remarkable resistance to argument and evidence” [46] include: “[f]ear of technological unemployment,” “self-concept” (perceptions of self-worth), “attachment to theory” (an idea that theory-mediated predictions do not contribute beyond what an atheoretical algorithm could produces cognitive dissonance), “misperception of the actuarial method as dehumanizing to clients or patients”, “general dislike of computers’ successfully competing with human minds,” and “poor education” [46]. On the topic of “poor education”, Grove & Meehl [46] elaborate:

Poor education is probably the biggest single factor responsible for resistance to actuarial prediction; it does not involve imputation of any special emotional bias or feeling of personal threat. In the majority of training programs in clinical psychology, and it is surely as bad or worse in psychiatry and social work, no great value is placed upon the cultivation of skeptical, scientific habits of thought; the role models—even in the academy, more so in the clinical settings—are often people who do not put a high value upon scientific thinking, are not themselves engaged in scientific research, and take it for granted that clinical experience is sufficient to prove whatever they want to believe.

Meehl & Grove [46] ultimately conclude their discussion with the following appeal to policymakers:

[P]olicy makers should not accept a practitioner’s unsupported allegation that something works when the only warrant for this claim is purported clinical experience. Clinical experience is an invaluable source of ideas. It is also the only way that a practitioner can acquire certain behavioral skills … [but] … [i]t is not an adequate method for settling disputes between practitioners, because they each appeal to their own clinical experience. . . . All policy makers should know that a practitioner who claims not to need any statistical or experimental studies but relies solely on clinical experience as adequate justification, by that very claim is shown to be a nonscientifically minded person whose professional judgments are not to be trusted. . . . To use the less efficient of two prediction procedures in dealing with such matters is not only unscientific and irrational, it is unethical. To say that the clinical-statistical issue is of little importance is preposterous.

What is interesting is the reactions of clinicians even *after* the evidence had mounted and was generally incontrovertible. This phenomenon is demonstrated by the Evidence Based Medicine (EBM) movement initially introduced in the 1990s.

On November 4, 1992, the Evidence-Based Working Group, chaired by Gordon Guyatt, published a consensus article describing the newly coined term *evidence-based medicine* as “a new paradigm for medical practice” [51]. The working group explained that “[e]vidence-based medicine de-emphasizes intuition, unsystematic clinical experience, and pathophysiological rationale as sufficient grounds for clinical decision making” [51]. The core concept of EBM was to transition clinical decisions from relying solely on a subjective, experience-based foundation to a more objective, evidence-based foundation by emphasizing the integration of research evidence and recognition of uncertainty into the scheme of clinical decision making [51]. Over the next two decades, the medical community slowly began to embrace the EBM paradigm as the standard of clinical practice. Today, nearly every medical school teaches EBM principles and numerous textbooks and journals have become devoted specifically to the topic creating in a new generation of physicians for which EBM is the default method for clinical practice. Looking back, it is considered by many “difficult to exaggerate the impact of EBM on the medical world” [52].

Although EBM ultimately became the standard for clinical practice, it was not without resistance and criticism—oddly reminiscent to the reactions from clinicians in years prior. Originally termed “scientific medicine”, Dr. Guyatt recounts the unsympathetic responses by many of his colleagues—“Those already hostile were incensed and disturbed at the implication that they had previously been ‘unscientific’.” [53]. Although the EBM moniker was more palatable, clinicians were openly disparaging and critical—particularly on the role of experienced-based opinions in the realm of the new paradigm [54,55]. The source of this criticism was often thought to be brought on by those within the profession looking to preserve autonomous, professional jurisdiction of decision making [54]; however, it highlighted the importance of a synergy between evidence-based principles and individual judgment. This was considered particularly important in situations where the context of an individual case is under-represented in the statistical data [56] and therefore insufficient to provide a meaningful contribution to the decision. Although EBM supporters recognize the value and necessity for individual judgment, it remained a point of debate as to how experience should be integrated with EBM evidence [56].

The EBM movement provides an important, and more recent, case-study illustrating the challenges with introducing algorithms into a domain that has traditionally been driven by human judgment. By the time EBM was initially introduced in the mid-1990s, the debate concerning the general superiority of algorithms was well established and the evidence was heavily favoring algorithms and statistical methods as superior to intuition and experience across a number of different domains. However, clinical practitioners continued to be reluctant to embrace it. Even more, the reasons cited for the resistance were not novel or specific to a unique aspect of EBM. On the one hand, these reactions can seem irrational, unscientific, and unethical from the perspective of proponents to the new paradigm (e.g., consider the responses by Grove & Meehl [46] in the discussion above). On the other hand, these reactions suggest the issue is more complex. As irrational as it might seem (to choose an inferior method between two options), there is clearly more to consider in order to understand *why* people tend to be averse to algorithms and *how* it can be overcome. In the section that follows, we take a step back and explore the dynamics of human—algorithm interactions more generally from perspectives of psychology and behavioral sciences to consider the tendency for people to rely on algorithms and factors that are believed to increase or decrease those tendencies.

### 1.2 Part II: Human—algorithm interaction in laboratory studies

As concepts and applications in artificial intelligence and machine learning bolstered the prominence of algorithms in the 1990s, it became clear that in order for the superior capabilities of algorithms to be realized, people had to be willing to rely on them. This phenomenon in which people tend to remain resistant to using algorithms and, when given the choice, often opt to rely on predictions made by a human compared to a superior algorithm—ultimately dubbed *algorithm aversion*—became an important focal point of research in psychology and human behavior. Rather than continually demonstrating the superiority of algorithms, the emphasis shifted to understand *why* people tend to be averse to algorithms and factors that may increase or decrease that tendency. In 2014, Dietvorst et al. note that prior literature on this topic has been limited to anecdotal experiences given the dearth of empirical evidence [57]. In an influential study involving incentivized forecasting tasks, Dietvorst et al. find that algorithm aversion, in part, hinges on people’s experience with the algorithm [57]—specifically:

[S]eeing algorithms err makes people less confident in them and less likely to choose them over an inferior human forecaster. This effect was evident in two distinct domains of judgment, including one in which the human forecasters produced nearly twice as much error as the algorithm. It arose regardless of whether the participant was choosing between the algorithm and her own forecasts or between the algorithm and the forecasts of a different participant. And it even arose among the (vast majority of) participants who saw the algorithm outperform the human forecaster.

Dietvorst et al. note that the resistance to algorithms is, at least partially, due to a greater intolerance for error from algorithms than from humans and that “people are more likely to abandon an algorithm than a human judge for making the same mistake.” [57]. Although these studies do not provide clear insights into how algorithm aversion can be overcome, the findings suggest proposed solutions will need to consider how to counter the apparent tolerance imbalance.

Following the work by Dietvorst [57], Logg et al. suggests the concept of algorithm aversion is not as straightforward as prior literature suggests and highlights boundary conditions for empirical evidence supporting algorithm aversion [58]. They argue that people are not necessarily universally averse to algorithms (particularly, prior to receiving any performance accuracy feedback); rather, they suggest that there is more to unpack on this topic and reliance on algorithms is likely to depend on several factors, such as *who* is relying on *which* advice and for *what* purpose [58]. For basic prediction tasks by lay people, such as non-incentivized numerical predictions based on visual stimuli (e.g., predicting the weight of an individual from a photograph) and forecasts about the popularity of songs and romantic attraction, Logg et al. show that “lay people adhere *more* to advice when they think it comes from an algorithm than from a person”—an effect coined as *algorithm appreciation* [58]. They note, however, that algorithm appreciation decreased when people chose between an algorithm’s estimate and their own (versus a different human judge) and when the people had expertise in the domain [58]. These findings suggest observations related to algorithm aversion may have also been bolstered by people’s excessive appreciation of their *own* opinions—a phenomenon well established in the literature and referred to as “overconfidence bias”—which has demonstrated that individuals treat their judgment as superior to that of others [58]. Further, and perhaps more concerning, they find that individuals bearing domain expertise were *least* likely to recognize the value of algorithmic advice [58]. Specifically, Logg et al. state: “Paradoxically, experienced professionals, who make forecasts on a regular basis, relied less on algorithmic advice than lay people did, which hurt their accuracy. . . . These results might help explain why pilots, doctors, and other experts are resistant to algorithmic advice. Although providing advice from algorithms may increase adherence to advice for non-experts, it seems that algorithmic advice falls on deaf expert ears, with a cost to their accuracy.” [58].

The findings by Dietvorst et al. [57] and Logg et al. [58] are significant in that they demonstrate that challenges persist in present day related to people’s willingness to rely on algorithms in certain conditions, despite general familiarity and presence of algorithms across nearly every industry. Although Logg et al. did not explicitly note the findings from Arkes et al. [59] regarding the impact of expertise three-decades prior, their observations were remarkably consistent and equally alarming. In their paper Arkes et al. [59] expanded on the popular *clinical vs. statistical* debate in the 1980s and provided one of the first empirical studies regarding specific conditions impacting people’s willingness to rely on algorithms vs. human judgment for prediction tasks. In a series of experiments, Arkes et al. evaluated willingness to rely on an algorithm (i.e., a simple classification rule) versus human judgment when three different conditions were manipulated: incentivization, instructional warning, and expertise [59]. Arkes et al. made three important observations: (1) “incentive for high performance resulted in less use of the decision rule whether the incentive was given for each correct judgment or for the best performance among a group of judges … [which] actually resulted in poorer performance”, (2) “warning subjects of the counterproductive results of abandoning the rule caused the subjects to use the rule more”, and (3) “those with expertise (or those who judged themselves to have expertise) were less likely to use a decision rule than those with less expertise … [and] [b]y choosing not to use the rule, such ‘experts’ performed worse but had higher confidence in their performance than the nonexperts.” [59]. They note (as did Logg et al. [58]) the likely impact of overconfidence bias as a cause for experts being less willing to rely on the algorithm decision aid. Specifically, Arkes comments: “One of the dangers of overconfidence is that one feels that no assistance is needed. If one assumes that his or her judgment is quite good, decision aids would be entirely superfluous. Indeed, in [our experiment] the more knowledgeable subjects were less likely to use the rule, which resulted in inferior performance.” [59]. Arkes et al. conclude with an important implication of these observations: “Note that in both the psychological and medical diagnosis scenarios described above, there exist well-meaning diagnosticians with high motivation, high expertise, and few constraints on innovative tendencies [e.g., lack of discipline to adhere to a decision rule]. These are the conditions under which decision aids are less likely to be used—to the detriment of those being served.” [59].

In 2016, as a follow-up study to their initial observations related to algorithm aversion (the phenomenon that people often fail to use evidence-based algorithms *after* learning that they are imperfect), Dietvorst et al. investigate strategies to reduce algorithm aversion by allowing people to exert some influence over the algorithm output [60]. Dietvorst et al. hypothesize that “[i]f people’s distaste for imperfect algorithms is in part driven by an intolerance of inevitable error, then people may be more open to using imperfect algorithms if they are given the opportunity to eliminate or reduce such errors. Thus, people may be more willing to use an imperfect algorithm if they are given the ability to intervene when they suspect that the algorithm has it wrong.” [60]. In evaluating this, Dietvorst et al. recognize that “[a]lthough people’s attempts to adjust algorithmic forecasts often make them worse, the benefits associated with getting people to use the algorithm may outweigh the costs associated with degrading the algorithm’s performance.” [60]. In a series of incentivized (non-expert) forecasting tasks in which participants could choose between using their own judgments or forecasts of an algorithm built by experts, Dietvorst et al. observe evidence supporting their hypothesis: “Participants were considerably more likely to choose to use an imperfect algorithm when they could modify its forecasts [even after seeing it err] … [and] … the preference for modifiable algorithms held even when participants were severely restricted in the modifications they could make.” [60]. Further, Dietvorst et al. note that participants who were able to modify the imperfect algorithm’s forecasts “reported higher satisfaction with their forecasting process and thought that the algorithm performed better relative to themselves compared with participants who could not modify the algorithm’s forecasts.” [60]. In closing, Dietvorst note that participants’ intervention “did often worsen the algorithm’s forecasts when given the ability to adjust them. However, we may have to accept this error so that, overall, people make less error.” [60].

The discussion above provides, in our view, important insights into the complex relationship between human—algorithm interactions. Despite the abundance of evidence in these disciplines demonstrating algorithms generally outperform humans, people tend to discount them in favor of their own judgments—even when their own judgments are known to be inferior—often resulting in lower accuracy than relying on the algorithm alone [46,47]. This phenomenon is most pronounced when the individuals have a high motivation to be accurate and possesses domain expertise in the prediction task (e.g., physicians performing medical diagnoses) [58,59]. Some researchers point to several sociopsychological factors based on anecdotal observations as potential causes [46]; others suggest it is a manifestation of overconfidence bias [58,59]. More recently, researchers found that this phenomenon is exacerbated when people are presented with the performance of the algorithm, and thus, the inevitable susceptibility to err, which often results in worse performance and impacts to business and society can be costly [57]. In an effort to explore solutions to mediate the impacts of algorithm aversion and increase the likelihood people are willing to rely on algorithmic advice, Dietvorst et al. find that allowing people to intervene and modify the algorithm’s output, even under limited conditions, tend to result in higher satisfaction, greater belief in the superiority of the algorithm, and higher likelihood to commit to using algorithms in subsequent tasks [60]. In a general sense, these observations illustrate an interesting paradox: to reduce error, we may need to accept error.^1^ Allowing for even just the *potential* for such intervention, despite constrained circumstances, appears to cater to people’s desires to incorporate their own judgments and *feel* they have some control over the outcome, thus resulting in a higher likelihood for adoption. Consequently, people are likely to be more satisfied with the process and performance will likely increase overall compared to the alternative in which people are more prone to reject the algorithm altogether in favor of their own judgments. Dietvorst et al. [60] note the operational implications of these findings and suggest:

[F]raming the decision of whether or not to use an algorithm as an all-or-nothing decision is likely to be counterproductive. People are unlikely to commit to using an algorithm’s forecasts exclusively after getting performance feedback or learning that it is imperfect. Furthermore, forcing employees into a regime in which they have to use an imperfect algorithm’s forecasts exclusively may lead them to become dissatisfied or push for a change. However, asking people to commit to an algorithm’s forecasts that they can modify by a limited amount seems much more palatable. People will be much more likely to choose to use an imperfect algorithm if they can modify its forecasts, and employees will not necessarily be dissatisfied if they are partially constrained to an imperfect algorithm’s forecast. . . . If for some reason having employees making constrained adjustments to an algorithm’s forecasts is not possible, [our study] shows that having employees make unconstrained adjustments to an algorithm’s forecasts can also substantially improve their forecasting performance.

These findings and recommendations have implications across a broad array of domains which are faced with increasing human—algorithm interactions, particularly in those domains traditionally dominated by human judgment (based on expertise and experience) and for which there is high motivation to be accurate. The empirical evidence is telling; however, what is more interesting is to reflect on human—algorithm interactions in distinct yet relevant real-world circumstances involving these conditions in context of these findings. For this purpose, we look at how things have unfolded in medicine as well as how things are presently unfolding in autonomous driving—a domain that *everyone* can relate to. These examples allow us to explore how people and society have grappled with the dynamics of human—algorithm interactions in these contexts and what seems to have ultimately proven to be successful (or at least palatable) over time. Indeed, as we discuss in the next section, it seems that irrespective of the specific domain, allowing human—algorithm *integration* is associated with an increase in people’s initial willingness to consider algorithms enabling them to ultimately grow more trusting, reliant, and accepting of the algorithms to influence the decision outcome.

### 1.3 Part III: Human—algorithm integration in real-world domains

In medicine, we have had the luxury to look back onto two (somewhat overlapping) eras and see how the issues have played out over time. As the evidence mounted demonstrating the superiority of algorithms, the idea that it was a dichotomous choice of one or the other was quickly met with resistance and criticism. Simply put, for various cited reasons (e.g., see Refs. [46,49,50]), clinicians were not willing to yield their clinical decision-making responsibilities to an algorithm—even though algorithms have been shown to provide superior performance. Over time, however, as the debates ensued, clinicians began to trend toward an integrated approach to bring algorithms into their scheme of clinical decision making (as opposed to wholesale outsource which often resulted in wholesale rejection) and the concept of “clinical *and* statistical” was introduced and slowly emerged as a workable solution [61]. Around that same time, when EBM was initially introduced in 1992, the authors of the Working Group stressed the importance of integrating research evidence into the decision-making scheme. At the outset, EBM was not presented as a dichotomous choice, but many clinicians clinging to the value of clinical judgment still reacted with criticism and outright rejection as if it were (see EBM 25–27]). Once again, the importance of judgment and experience in the scheme of clinical decision making was highlighted by those concerned it would go to the wayside. Although viewpoints were polarized, as the initial reactions subsided, clinicians naturally trended toward a reasonable middle ground and clinical decision making became an integrated and multi-faceted approach of “clinical *and* statistical”. As Coen et al. highlighted: “Perhaps EBM should be renamed ‘methods of incorporating epidemiologic evidence into clinical practice’ … but this is quite a cumbersome moniker.” [56].

Looking retrospectively, we see that within the domain of medicine, what started out as a “clinical *versus* statistical” debate naturally transitioned into a “clinical *and* statistical” integrated solution. Interestingly, one may argue that the present-day research related to human—algorithm interactions offers a reasonable explanation. Indeed, physicians possess high expertise, are highly incentivized and motivated to provide accurate decisions, and operate fairly autonomously. As noted previously: these are the conditions under which people are most likely to rely on their subjective judgment and least likely to accept algorithms [58,59]. The solution proposed by Dietvorst et al. [60] appears to have naturally taken shape. By structuring the scheme of clinical decision making as an integrated approach based on statistical evidence and subjective judgment, the clinician maintains the ability to exert some influence on the overall outcome—whether that is by adjusting for idiosyncratic factors that are shown to be under-represented by the statistical evidence or by relying on the statistical evidence as an additional pillar to support the overall foundation of the decision. While this approach seems to provide the conditions that are most appealing for practitioners in terms of their willingness to adopt, there is concern that clinicians are too quick to find “exceptions” in the statistical data and adjust in favor of their subjective judgment [62]. How those “exceptions” can and should be moderated remains an open question.

In autonomous driving, the issues of human—algorithm interaction can be viewed through the public’s willingness to embrace automation to either supplement or supplant their own driving tasks—tasks for which drivers generally consider themselves to have expertise based on specialized knowledge and experience and for which there are high stakes and serious safety concerns for inaccurate decisions. For background, in 2014, SAE International first published the standard J3016 Levels of Driving Automation which defined six levels of vehicle automation ranging from Level 0 (no automation) to Level 5 (full automation), transitioning gradually from “driver support features” to “automated driving features” [63]. The SAE J3016 Levels of Driving Automation taxonomy was subsequently adopted by the United States National Highway Traffic Safety Administration (NHTSA) in 2016 as a formal taxonomy for describing increasing levels of automation and shifting roles from the human to machine for executing dynamic driving tasks. For context, the levels of the J3016 standard are: Level 0 (No Automation), Level 1 (Driver Assistance), Level 2 (Partial Automation), Level 3 (Conditional Automation), Level 4 (High Automation), Level 5 (Full Automation) [63]. In levels 0 through 2, the human maintains full control with increasing assistance from technology and in levels 3 through 5, the system is in control with decreasing need for human intervention [63]. The levels codified by the J3016 standard provide a useful framework for considering people’s willingness to engage in progressive levels of vehicle automation and shifting responsibility and control from human to machine for various driving tasks.

First, from the perspective of safety it is important to consider the necessity of moving toward automation in consumer vehicles. For example, in 2016, the United States Department of Transportation released the NHTSA fatal traffic crash data on American roadways and the results were startling: human choices were linked to 94% of serious crashes resulting in a call to “promote vehicle technologies [to] … help reduce or eliminate human error and mistakes that drivers make behind the wheel” [64]. These results alone demonstrate that the case for vehicle automation is clear and people should embrace automation with open arms—after all, it will improve safety and save lives. However, once again, they often *don’t*. In 2016 and 2017, surveys were conducted regarding consumer interest in automation and the highest level of automation in vehicles they would be willing to consider. In 2016, Shoettle and Sivak analyzed 618 survey responses from participants throughout the United States and found that 45.8% of respondents preferred no self-driving capabilities, 38.7% preferred partially self-driving capabilities, and only 15.5% preferred full self-driving capabilities [65]. When asked about the preferences for controlling completely self-driving vehicles, Shoettle and Sivak found that “[n]early all respondents (94.5%) would want to have a steering wheel plus gas and brake pedals (or some other controls) available in completely self-driving vehicles [65]. In 2017, Abraham et al. released a follow-up to a survey completed in 2016 to explore changes in perception from one year prior [66]. This was due in large part because shortly after the initial data were collected from the 2016 survey, the world saw the first fatality related to a highly automated driving feature [67]. In their follow-up survey, Abraham et al. analyzed 2976 survey responses from participants throughout the United States and found “a significant decrease in the proportion of respondents who were comfortable with the idea of a fully self-driving car and an apparent shift toward more limited automation in the form of ‘features that actively help the driver while the driver remains in control.’ Similarly, there was a proportional decrease in those who were comfortable with features that periodically take control of driving.” [66]. Further, Abraham et al. found that among participants who reported they would never purchase a self-driving car, “[t]he most cited hesitation was discomfort with the loss of control; other commonly mentioned factors included not trusting the technology, a disbelief that it would be robust enough to rely on exclusively, and a feeling that self-driving cars are unsafe.” [66]. Abraham et al. conclude with the following [66]:

The perception that self-driving cars need to work perfectly to be acceptable, combined with present and past experiences of low-risk technology failure both in and out of vehicles, may lead many consumers to believe the technology will never be good enough such that they can trust it with their lives. The difficulty here is that it remains an open question as to how safe a self-driving vehicle needs to be in order to become socially acceptable as a mobility option. . . . Encouraging the appropriate use of driver assistance and other human-centric automated vehicle systems by investing in educational resources that consumers prefer may be an important stepping stone to improving consumer interest, confidence, and trust in self-driving technology.

Around this same time, in 2017 the RAND Corporation released their report “The Enemy of the Good: Estimating the Cost of Waiting for Nearly Perfect Automated Vehicles,” which explored this very issue [68]. The RAND Corporation open their report with the current quandary:

[A] key question for the transportation industry, policymakers, and the public is how safe [highly automated vehicles (HAVs)] should be before they are allowed on the road for consumer use. From a utilitarian standpoint, it seems sensible that HAVs should be allowed on U.S. roads once they are judged safer than the average human driver so that the number of lives lost to road fatalities can begin to be reduced as soon as possible. Yet, under such a policy, HAVs would still cause many crashes, injuries, and fatalities—albeit fewer than their human counterparts. This may not be acceptable to society, and some argue that the technology should be significantly safer or even nearly perfect before HAVs are allowed on the road. Yet waiting for HAVs that are many times safer than human drivers misses opportunities to save lives. It is the very definition of allowing perfect to be the enemy of good. . . . The lack of consensus on how safe HAVs should be before they are allowed on the road for consumer use reflects different values and beliefs when it comes to humans versus machines.

To explore this issue further, the RAND Corporation conducted a series of analyses comparing road fatalities over several decades under different theoretical policies in which HAVs are deployed when they are just 10% better than the average human driver (*Improve10*) or wait until they are 75% better (*Improve 75*) or 90% better (*Improve90*) than the average human driver. From these analyses, the RAND Corporation found [68]:

In the short term (within 15 years), more lives are cumulatively saved under a more permissive policy (Improve10) than stricter policies requiring greater safety advancements (Improve 75 or Improve90) in nearly all conditions, and those savings can be significant—hundreds of thousands of lives. The savings are largest when HAVs under Improve10 are adopted quickly. . . . In the long term (within 30 years), more lives are cumulative saved under an Improve10 policy than either Improve75 or Improve90 policies under all combinations of conditions we explored. Those savings can be even larger—in many cases, more than half a million lives.

These data demonstrate the value of moving toward vehicle automation sooner rather than later. Despite these findings, people remain in disbelief and reluctant to accept the technology. In their review of the literature related to consumer acceptance of automated vehicle technology between 2013 and 2019, Jing et al. note that despite the rapid development of the technology, public acceptance of automated vehicles is one of the major factors affecting widespread distribution. Specifically, the term “safety” was the most frequently occurring word in all of the collected literature: “Some respondents even estimate autonomous driving is not as safe as human driving. Hence, they are more willing to accept [automated vehicles (AVs)] with manual driving options than fully AVs without steering wheels. The deaths of AV accidents reported in recent years may intensify public suspicion about the safety issues, and safety concerns have proven to be a potential deterrent to the acceptance of AVs.” [69].

The issues concerning autonomous driving once again illustrate the pervasive impact of people’s reluctance to accept imperfect algorithms and disjointed expectations that algorithms need to be nearly perfect before accepting them. Ironically, despite the evidence that automation in vehicles is *more*-safe, the most commonly cited reason for peoples’ hesitation to adopt is due to concerns that they are *less*-safe. The barriers to improved safety and performance are, once again, rooted in peoples’ reluctance to rely on the algorithms—particularly after news of an accident where the technology was involved. Despite the apparent aversion, the evidence shows that people will be more willing to accept autonomous vehicles *if* they still have the option to maintain control and can rely on their own judgment and decisions [65]. Within the domain of vehicle automation we see again that the findings from Dietvorst et al. appear to generalize well—people are averse to relying on algorithms after seeing them fail (even though the algorithms’ overall performance is better than human judgment alone) and people tend to hold algorithms to higher standards than their human counterparts, demanding near perfect performance before personally embracing them [57]. Further, we see that allowing the human to have some control over the outcome of the driving task tends to increase their willingness to work *with* the algorithm—a possible solution to reduce the effects of algorithm aversion proposed by Dietvorst et al. [57].

Human—algorithm interactions in both medicine and autonomous vehicles are not too different from one another, despite the apparent orthogonal relation of the two domains. The issue ultimately boils down to trust and confidence with the algorithms. People naturally trust themselves and their *own* judgment (however flawed it may be) over other sources, particularly when they are the ones ultimately responsible for the outcome or have some inherent incentive to be accurate. By introducing increasing levels of automation designed to *supplement* the human as opposed to immediately *supplant* the human, people tend to be more willing to incrementally accept the increased intervention of automation and slowly become more comfortable and trusting in the technology. As comfort and trust in the technology evolves, reliance on the algorithms will increase resulting in improved performance and safety over time.

### 1.4 Part IV: Algorithms and the american legal system

As algorithms have advanced and automated decision systems have become more accessible, researchers, advocates, and policymakers are debating when and where these systems are appropriate—including particularly sensitive domains such as criminal justice [70]. Questions have been raised on how to fully assess the short and long-term impacts of these systems and the appropriateness of their applications given many operate as “black-boxes” [70]. In an effort to keep pace with these types of issues, the first G7 Multi-stakeholder Conference on Artificial Intelligence was held in Montreal, Canada in December 2018 with the overarching theme of “Enabling the Responsible Adoption of AI” [71]. Over 200 experts in artificial intelligence (AI) attended the conference, representing all of the G7 countries and beyond, as well as key multi-stakeholder perspectives from industry, academia, civil society, and government. Among those in attendance was Geoff Hinton, world-renowned computer scientist, industry leader in AI, and developer of the “Google Brain”. During an interview at the conference, when prompted about AI’s eventual role in decision making, Hinton responded [72]:

I’m an expert on trying to get the technology to work, not an expert on social policy. One place where I do have technical expertise that’s relevant is [whether] regulators should insist that you can explain how your AI system works. I think that would be a complete disaster.

People can’t explain how they work, for most of the things they do. When you hire somebody, the decision is based on all sorts of things you can quantify, and then all sorts of gut feelings. People have no idea how they do that. If you ask them to explain their decision, you are forcing them to make up a story.

Neural nets have a similar problem. When you train a neural net, it will learn a billion numbers that represent the knowledge it has extracted from the training data. If you put in an image, out comes the right decision, say, whether this was a pedestrian or not. But if you ask “Why did it think that?” well if there were any simple rules for deciding whether an image contains a pedestrian or not, it would have been a solved problem ages ago.

In a follow-up question, when asked about how should people trust these algorithms, Hinton responded [72]:

You should regulate them based on how they perform. You run the experiments to see if the thing’s biased, or if it is likely to kill fewer people than a person. With self-driving cars, I think people kind of accept that now. That even if you don’t quite know how a self-driving car does it all, if it has a lot fewer accidents than a person-driven car then it’s a good thing. I think we’re going to have to do it like you would for people: You just see how they perform, and if they repeatedly run into difficulties then you say they’re not so good.

Hinton’s remarks during this interview were immediately met with criticism, challenging the notion that scientists working to develop algorithms can separate themselves from downstream implications resulting from algorithm applications. For example, Dr. Heather Roff from the University of Cambridge responded [73]:

This is a dangerous position to take. An expert on technology who feels themselves divorced from social or policy implications does not understand that technology is not value neutral, and that their decisions—even seemingly basic ones on how many gradient descents to take in a system—have socio-political implications. If one thinks they are only Scientists doing Science, but then simultaneously think that regulators should take an interest has fundamentally misunderstood their role as scientists engaging in socially and morally important questions. If your work requires legislation then you should think about that at the design stage … period.

As illustrated by the exchange above, it would be a naïve viewpoint to consider the issue of algorithm implementation in a specific domain complete without consideration of the environment for which the algorithms are ultimately applied and the implications of such applications. Within the broader criminal justice context, law enforcement leaders are strategizing how to leverage the benefits that algorithms provide within various aspects of policing and criminal justice, but in doing so have stressed the importance of maintaining public trust and upholding societal values by ensuring algorithms are characterized by fairness, accountability, transparency, and explainability [[74], [75], [76]]. In the case of forensic science, an important consumer of the forensic results is the legal system, which bears the ultimate responsibility for ensuring all people receive fair and equitable justice under the law. Although algorithms have demonstrated remarkable potential to provide advanced scientific capabilities and promote objective foundations to the ultimate issues in question, they do so often at the cost of transparency and explainability [[77], [78], [79], [80], [81], [82], [83]]. In some cases, algorithms may operate as a black-box due to trade secrets or other legal protections asserted by the manufacturer. In others, they may manifest as a black-box due to their computational complexity. In either situation, legal actors have expressed concern that the opacity of algorithms can stifle meaningful scrutiny and accountability of the systems thereby infringing on criminal defendants’ Constitutional rights [see 77–78, 80–81]. These issues are exacerbated by examples in which algorithms have indeed perpetuated historic inequities (e.g., see Refs. [70,82,84,85]). Faced with these concerns, courts have found themselves arbitrating complicated legal questions forcing them to grapple with issues concerning the admissibility of algorithms and their implications to the law. Legal scholars have begun to explore these issues in various contexts within the American legal system—most notable and relevant to our discussion are those by Imwinkelried [77] and Nutter [81], which are briefly summarized below. Our intent here is to be illustrative, not exhaustive, of the importance to consider broad downstream legal implications of algorithms when deciding when and how to apply them to a particular (and sensitive) domain, such as forensic science for criminal justice purposes. Specific technologies and circumstances concerning their applications within the criminal justice pipeline may create additional implications and it would be impractical to cover them all in this discussion. Our discussion is intentionally generic in terms of the specific legal issues and narrowly focused on the application of algorithms developed for purposes of augmenting traditional forensic science methods which rely predominantly on human judgment and expertise. Although we borrow examples from probabilistic genotyping for illustrative purposes, our focus is directed toward pattern and impression evidence disciplines and is not meant to apply to all types and applications of algorithms that have been, or could be, introduced into litigation. Finally, we do not consider the issue to be *whether* algorithms should be implemented into forensic practice for criminal justice purposes—we consider the issue to be *how* to implement them in a way that is cognizant of the legal issues and increases the likelihood legal stakeholders will be willing to consider them within their own regulatory framework.

The legal issues concerning the application of algorithms to pattern and impression evidence has yet to be fully explored. Only recently legal scholars begun to unpack the issues and consider how the legal system can adapt to the inevitable application of algorithms while maintaining their gatekeeping function. In 2016, Imwinkelried considers the issue in the context of the expanded use of algorithms for probabilistic genotyping software introduced in 2009 using TrueAllele software from Cybergenetics, Inc [77]. Probabilistic genotyping software analyzes DNA mixtures and provides a statistic that helps assess whether or not a particular defendant was one of the contributors. Although TrueAllele is not the only probabilistic genotyping software available, it has received attention in the United States due to its use and the manufacturer’s assertion of trade secret protections when criminal defendants have requested its source-code to examine its reliability. Ultimately, courts have largely rejected defendant’s requests for disclosure and independent review of source-code and ruled in favor of admissibility, which has led to an outcry by defense litigators (see Refs. [[77], [78], [79], [80]]). After reviewing prior case law admitting testimony based on technologies such as TrueAllele while denying defendants access to the source-code, Imwinkelried presents a critical analysis of the legal issue. In particular, Imwinkelried addresses the question of whether the prosecution should be permitted to introduce expert testimony based on a computerized technique without presenting foundational testimony about the validity of the program’s source code controlling the technique and, if so, whether there are circumstances in which the defense ought to have access to the source code despite the trade secret assertions protecting such disclosure [77].

The first issue considered by Imwinkelried is the admissibility of a computerized technique without providing foundational testimony about the validity of the source-code controlling the technique. In other words, whether the evidence produced by the system should be admitted without first revealing the underlying code controlling the operation of the algorithm and the algorithm itself. In the United States, a minority of non-federal jurisdictions still rely on the 1923 *Frye* standard of “general acceptance” [86]. Under this standard, the proponent need not demonstrate foundational validity at all; rather, as Imwinkelried describes, the proponent only needs to demonstrate whether the “theory or technique has gained a certain degree of popularity—’general acceptance’—within the relevant scientific fields.” [82]. In 1975, however, the Federal Rules of Evidence took effect, which led to the 1993 ruling in *Daubert v. Merrell Dow Pharmaceuticals, Inc.* [87], the first of a trilogy of Supreme Court decisions on the admissibility of expert testimony (*Daubert v. Merrell Dow Pharmaceuticals, Inc.* [1993] [87]*,* General *Electric Co. v. Joiner* [1997] [88], and *Kumho Tire Co. v. Carmichael* [1999] [89]—collectively referred to as the “*Daubert* standard”). Under the *Daubert* standard, the proponent must demonstrate that the theory or technique rests on adequate validation for which trial judges bear that gatekeeping responsibility. Today, federal and the majority of non-federal jurisdictions rely on the *Daubert* standard as a framework for admissibility. It is under this standard that many criminal defendant’s assert that a computerized technique, without access to the underlying source-code and algorithm itself, should be inadmissible due to the inability to demonstrate its validity. However, as Imwinkelried describes, courts have ruled that the burden of demonstrating the validity of a technique can be met “by presenting testimony about the validation studies investigating the accuracy of the software. . . . [t]he very purpose of a validation study is to investigate whether the theory or technique does what its proponent claims.” [77]. Imwinkelried argues (in the context of TrueAllele) [77]:

Federal Rule of Evidence 901(b)(9) [90] captures the essence of the “authentication” or validation of a scientific technique. In the words of 901(b)(9), the essential foundation is a “showing that [the process or system] produces an accurate result.” [citing 90]. Validation studies summarizing the results of tests of the technique and showing that the technique yields accurate results satisfy that standard. As a matter of logic, the court should treat the studies as adequate validation under Daubert. The proponent can shoulder the burden of Daubert without making a further, separate showing about the source code of the software controlling TrueAllele. The lack of testimony about the source code might increase the degree of uncertainty in the expert’s final opinion, but post-Daubert, the expert need not vouch for his or her opinion as a certainty. In short, many courts have reached the correct result that prosecution evidence based on TrueAllele can be admitted, even without testimony about the source code.

Ultimately, Imwinkelried argues that, in the context of probabilistic genotyping software in particular, courts have rendered appropriate decisions from a legal standpoint as to the admissibility of algorithms without the requirement that the source-code (and algorithm thereto) be released [77].

Admissibility, however, is only one of the legal issues to consider. As Imwinkelried notes, “[e]ven when the proponent’s item of evidence is admissible, the opponent has the right to attack the weight or believability of the evidence. . . .The U.S. Supreme Court has held that in criminal cases, the defendant’s right to attack the weight of the prosecution’s evidence is of constitutional dimension under the Sixth Amendment Confrontation Clause.” [77]. Indeed, when handing down the ruling, the *Daubert* court noted: “Vigorous cross-examination, presentation of contrary evidence, and careful instruction on the burden of proof are the traditional and appropriate means of attacking shaky but admissible evidence.” [87]. This leads to the second issue—in the absence of the underlying algorithm and source-code, are defendant’s deprived of the ability to challenge the credibility of the evidence? This question is more complicated. Although Imwinkelried recognizes that prior courts have pointed to the existence of validation studies as the means to enable opponents to evaluate the accuracy of the system, Imwinkelried also notes: “The answer does not turn on the mere existence of validation studies or even their availability to the defense. Rather, the answer depends on the number of studies, their quality, and a comparison between the test conditions and the conditions in the instant case.” [77]. Consequently, if these factors are not well established, it might seem that the source code is warranted; however, such a decision has to be considered in light of the countervailing argument of the proponent’s assertion of trade secret protections and “[f]aced with competing legitimate interests, a trial judge must attempt to strike a rational balance.” [77]. Ultimately, Imwinkelried proposes a judge could accomplish this by proceeding in two steps [77]:

First, a judge should assign to the accused seeking discovery the burden of showing that the facts of the instant prosecution exceed, or are at the margins of, the validation range of the empirical studies relied on by the prosecution. More specifically, the defendant must convince the judge that the available studies do not adequately address the effect of a specified, material variable or condition present in the instant case. The most clear-cut case would be a fact situation in which none of the available studies relied on by the prosecution experts tested the application of the technique to fact situations involving the condition. . . . [However], [t]he judge should certainly not accept the *ipse dixit* assertion of the defense counsel that the omitted condition is material in the sense that its presence could affect the outcome of the test. . . . Rather, the judge ought to demand that the defense present expert testimony explaining why it is plausible that that condition could change the test result.

Assume that in the first step, the judge concludes that the defense has met its burden. Even then the judge should not automatically require the manufacturer to furnish the defense with a printout or electronic version of the source code. Instead, the judge could give the manufacturer a choice to: either (1) allow the defense to test the application of the program to a fact situation including the material condition or variable omitted from the validation studies, or (2) provide the defense with the source code. . . . By enabling the defense expert to conduct a new validation study testing that application, the manufacturer would afford the defense expert a fair opportunity to investigate the merit of the criticism … [and ultimately] determine whether the inclusion of the additional condition could actually—not merely theoretically—affect the outcome of the use of the automated forensic technique.

Imwinkelried concludes: “Until courts guarantee the defense [the ability to challenge trustworthiness], source code will continue to be a source of controversy and doubt about the marked trend toward the automation of forensic analysis in the United States.” [77].

In 2019, Nutter considers the issues with an added layer of complication when the evidence is the product of machine learning algorithms [81]. The distinction is that the source code for machine learning algorithms is practically uninterpretable, even for the manufacturer. Thus, contrary to the prior discussion, disclosure of source code in this context would not materially advance the a party’s interests of ensuring the reliability of the algorithm. This discussion is relevant to illustrate the legal issues when the algorithm is truly a “black box” and a legal order for source code disclosure is not a practical solution. In the discussion below, although Nutter considers the issues in the context of general “machine learning algorithms”, the issues are analogously applicable to any algorithm that operates or manifests as a black-box (due to legal protections outside of the Court’s realm of control or due to computational complexities); thus, for purposes of this discussion, references to “machine learning algorithms” are considered synonymous with “black-box algorithms” in general.

Nutter addresses the issues of machine learning evidence (e.g., black-box algorithms) in criminal prosecution from a prospective standpoint, recognizing that it is only a matter of time until courts will be required to grapple with these issues. In doing so, Nutter “aims to look ahead to possible evidentiary issues when, not if, the output of machine learning algorithms is used as substantive evidence in criminal prosecution.” [81]. This context is particularly important as it could enable proponents of forensic algorithms to consider these issues *a priori* when the algorithms are developed and ultimately implemented into forensic practice in a way that recognizes the concerns from legal stakeholders and promotes judicial efficacy.

Like Imwinkelried [77], Nutter first explores the legal issues concerning evidence generated from machine learning algorithms from the perspectives of admissibility [81]. Both Nutter [81] and Imwinkelried [77] share similar perspectives on the issue of admissibility under Evidence Rule 702 [91] and *Daubert* [87]—although there is nothing inherently inadmissible, proponents of the algorithm will need to ensure the validation of the system is applicable to the circumstances of the existing case. However, Nutter takes the discussion a step further and also considers the implications of algorithms under the Constitution. Nutter notes that “[s]everal constitutional provisions may be implicated by machine learning identification in criminal prosecutions. Defendants may cite the Fifth Amendment’s Due Process Clause [92] or the Sixth Amendment’s Confrontation Clause [93]” under the premise of “guilt by black-box” [81]. Under such an argument, Nutter suggests defendants might claim the lack of transparency and explainability of how the algorithm arrived at the particular conclusion deprives the defendant the ability to challenge its credibility and disclosure of source-code is not the effective remedy [81]. On the basis of a Due Process argument, with reference to analogous past precedent, Nutter ultimately concludes that the Court would most likely “find due process satisfied when (1) the defendant can at least challenge the data that go into the algorithm (a requirement that can be addressed with procedural rules of discovery wholly within the Court’s control) and (2) the algorithm possesses some sufficient level of accuracy, which can come to light at a *Daubert* hearing on admissibility or cross-examination at trial.” [81]. Consequently, and furthermore, Nutter argues that “it is likely that the Sixth Amendment’s Confrontation Clause would require an expert to testify in-person and be subject to cross examination.” [81]. Taken together, Nutter ultimately suggests that despite the black-box nature of the algorithm, neither Constitutional provision will categorically bar machine learning evidence; however, the weight of such evidence may be impacted because of the machine learning’s distinct unexplainably rendering it difficult, if not impossible, to explain how the algorithm makes a particular conclusion [81]. It is this inherent unexplainably that will present the greatest challenge to proponents of algorithms that operate or manifest as a black-box—despite their admissibility.

Similar to Imwinkelried [77], Nutter recognizes that although there is nothing inherently inadmissible about black-box algorithms, questions remain regarding the weight of such evidence at trial [81]. Accordingly, there will be “considerable onus on trial counsel to persuade the trier of fact to discount the weight that the evidence should be assigned … [and] [j]urors might be cautious to assign much weight to machine learning evidence because of its peculiar property that it is often not explainable.” [81]. Nutter explains [81]:

It is an entirely open question the extent to which, in open court, jurors would trust the validity of unexplainable machine learning evidence. Indeed, this question is ripe for empirical research by psychologists and legal scholars of scientific evidence. Developers understand that the extent to which a person trusts a machine in everyday life is highly variable and context-dependent. Outside the courtroom, an individual’s trust in a machine ranges from none or little (for a variety of reasons, one of which is often because it is a machine [referencing 57]), to passive trust in machines without so much as a second thought. [Further] … [r]esearchers find trust in machines to be highly variable and influenced by different factors like belief about the functionality of the technology, belief that the technology is helpful, and belief that the technology is reliable. . . . Inside the courtroom, how jurors will respond to machine learning output is very difficult to predict. . . . Additionally, inextricably linked to the credibility of the machine is the credibility the jurors extend to the testifying expert him- or herself. That human credibility would likely affect credibility that jurors would extend to the underlying machine, especially as the scientific evidence at issue is particularly complex for laypeople. In that case, the prosecution or defense would surely already be familiar with the usual tactics to use to attack the expert’s credibility.

This last point raised by Nutter [81] brings us to our final point of concern when considering the issue of introducing algorithms into the legal system. From the discussion above, we see that the most significant issues are less about whether the algorithms would be admissible or not—provided they were adequately validated in a way that are representative of the circumstances for the case at hand, then they are likely to be found admissible. Rather, the issues are more so the extent to which fact-finders will be receptive of the evidence generated by the algorithm and afford it the appropriate weight. Thus, in addition to being considerate of issues that might be raised concerning admissibility, proponents of the algorithms will also need to concern themselves with factors that might increase or decrease jurors’ and judges’ willingness to trust the results of the algorithm (and by extension, human—algorithm combination). This, in turn, causes us to think about two additional issues and their implications to practice: (1) the extent to which the expert will need to be knowledgeable about the underlying algorithm and method employed when faced with such challenges, and (2) *how* the algorithm is implemented at the laboratory and used by the expert.

To expand further on the first point, the implementation of an algorithm will require more than a mere policy change. Such decisions will need to be accompanied by robust training and education to ensure experts are able to be responsive to questions and challenges raised during testimony. Implementation without proper education and training could detract from the overall credibility of the evidence and undermine the benefits it is intended to provide. The depth of that knowledge, however, may depend on how the algorithm is implemented and the extent to which the final conclusion was dependent upon the algorithm. To expand further on the second point, in some situations, such as probabilistic genotyping, the use of the algorithm is necessary to derive information that is otherwise difficult to interpret by the human; thus, the algorithms provide a capability that was otherwise non-existent. The output of the algorithm is the *sole basis* of the information. Laboratories (and experts) are much more limited in how they use the algorithms in these contexts and fact-finders have little choice but to rely on the algorithm or discount the information altogether. In other situations, such as traditional pattern evidence disciplines, the use of algorithms can be done in parallel with the human to assist with quantifying the value of impressions independent from the value assigned by human judgment alone. The output of the algorithm is a *supplemental basis* of the information. Thus, the algorithms could be applied to impressions that would normally be considered “no value” through subjective interpretation alone (e.g., see Ref. [94]) thus providing additional information for the courts to consider, *or* they could be applied to impressions for which experts believe have associative value through their subjective interpretation, but unable to substantiate empirically. It is this latter condition that we are particularly interested in exploring further since it characterizes the most immediate point of concern among scientific and legal scholars calling for algorithms—the need for empirical substantiation so that conclusions do not rely solely on human judgment [3,[7], [8], [9]].

In circumstances where the algorithm is not a precondition for interpretation, we have flexibility to consider different strategies for how an algorithm could be implemented within the broader examination methodology and the pros and cons of one approach over another—both in context of practitioners’ willingness to adopt the algorithm and fact-finders’ willingness to rely on evidence generated by the algorithm. In the section that follows, we discuss these issues further and ultimately propose a path forward for the implementation of algorithms into forensic practice that is believed to increase the likelihood for adoption across all stakeholders and lead to an overall stronger foundation and improvement to the quality and consistency of forensic science in general and pattern evidence examination in particular.

### 1.5 Part V: A path forward for forensic science

Over the years, several forensic science disciplines have been encouraged to adopt algorithms (i.e., statistical methods). The perceived benefits of algorithms are wide-ranging, but the immediate advantage (particularly for the pattern evidence domains) is to provide an empirical foundation to the evaluation of forensic evidence [3,[7], [8], [9]]. Although the calls for algorithms in forensic science have prompted researchers to propose numerous potential technology solutions, none have addressed the fundamental questions or strategies of how algorithms should be (or could be) implemented operationally. In the preceding discussions, we have explored the benefits algorithms provide as well as issues of human—algorithm interactions in several different ways. Collectively, these explorations have enabled us to characterize key challenges and consider strategies to reduce the barriers for algorithms to be implemented within forensic science. In this section, we consider *how*, not *if*, algorithms could be implemented into operational practice in such a way that forensic practitioners and other legal and scientific stakeholders are likely to accept. With the context of prior discussions in mind, we first explore different ways that algorithms could be implemented operationally within the examination methodology and implications of those approaches to future practice. Then, we outline a path forward for laboratories to consider as a strategy for implementing algorithms operationally and progressively moving toward ensuring evidence is presented with stronger scientific foundations.

In Parts I through III, we found that the implementation of algorithms into domains traditionally dominated by human judgment is often fraught with resistance [46,[48], [49], [50],^54^,^55^]. People tend to exhibit a general aversion to algorithms and prefer to rely on their own judgment—often despite knowledge that their own judgment is typically inferior to that of algorithms [57]. This phenomenon is exacerbated when people possess domain expertise [58,59], are faced with high-stakes decisions [58,59], and are presented with an algorithm that is susceptible to err [57]. Although the actual source of these reactions has not yet been fully understood, some researchers have pointed to various sociopsychological factors [46], overconfidence bias [58,59], and a general lack of trust in algorithms’ abilities to account for idiosyncratic factors [46] as possible explanations for the behaviors. Finally, both anecdotal observations of human—algorithm interactions in different domains and recent research have suggested that people tend to be more receptive to algorithms if they are integrated as a factor that *supplements* as opposed to *supplants* human decision making and the human retains some amount of influence on the ultimate outcome [60,61]. The above provides important context when considering the implementation of algorithms into forensic science. Indeed, forensic science has the major conditions for which algorithm aversion is most pronounced: (i) forensic examination results (in the pattern evidence domains particularly) are traditionally based entirely on subjective judgment, (ii) forensic examiners possess expertise, and (iii) forensic conclusions involve high-stakes decision-making. Thus, we have no reason to expect the reactions and behaviors of forensic practitioners to be substantially different than what has been observed in research and other domains explored. In fact, to some extent we have already observed similar behaviors manifest. Practitioners’ reactions to the mere notion of implementing statistical approaches have been met with criticism and opposition from practitioners [[38], [39], [40], [41]]. Further, when given the opportunity to incorporate algorithms into their decision-making, practitioners tended to disregard them in favor of their own judgments [95]. In an appendix to a discussion regarding the presentation of probabilities in a moot-court exercise related to fingerprint evidence, Langenburg addresses a list of fears that he has commonly heard from practitioners as he has traveled around various jurisdictions providing training [96]. The anecdotal reactions outlined by Langenburg [96] in the context of fingerprint examinations are eerily similar to those addressed by Grove & Meehl [46]. The comparison and recognition of similarities between non-forensic and forensic domains related to reactions to algorithmic interventions and human—algorithm interactions are important because they allow us to understand and be responsive to the perspectives of forensic practitioners and consider strategies for implementation such that practitioners might be more willing to embrace.

In addition to characterizing the anticipated concerns from forensic practitioners, we also need to be considerate of the needs of the legal system as it relates to the implementation of algorithms. Ultimately, the legal system is concerned with ensuring defendants receive fair and equitable justice under the law. Accordingly, courts will need to consider the admissibility of algorithms against existing legal standards and ensure they are used in a way that does not infringe on defendants’ Constitutional rights. In Part IV, we found that this can be particularly challenging given the “black-box” nature of many algorithms and, in some cases, the countervailing legal protections against disclosure of the actual algorithm and source-code. Defendants will often argue the opacity of algorithms fail to demonstrate reliability under Evidence Rule 702 and *Daubert* standards for admissibility. Further, defendants might claim that “black-box” algorithms deprive them of their Fifth Amendment right of Due Process and Sixth Amendment right of Confrontation. Ultimately, legal scholars have opined that algorithms are likely admissible under existing evidentiary rules and standards; however, (i) they will likely need to be introduced as part of expert testimony, (ii) experts will likely face challenges as a proxy to the algorithm, and (iii) the weight fact-finders give to the evidence could be impacted in unpredictable ways. At times, jurors may be more receptive to the evidence because it is the product of an algorithm. In others, jurors may be more skeptical because of their lack of trust and understanding of the system and deflection of any negative perceptions they may have of the creditability of the expert. Accordingly, experts will need to have sufficient familiarity with the algorithm and be able to answer to the challenges under cross examination. Additionally, besides testifying to the overall result, experts will need to be able to help educate fact-finders on issues related to the validation of the algorithm, conceptual operation of the algorithm, how the algorithm is factored into the overall examination methodology, and the extent and manner in which the algorithm influences the overall interpretation of the evidence. These details are important as they allow us to be responsive to the perspectives of legal stakeholders and consider strategies for implementation such that the legal actors are more willing to embrace.

From the above discussion, we see that the implementation of algorithms into many forensic science disciplines are likely to face considerable headwind from practitioners and will require careful consideration of the legal issues and resulting implications. These anticipated challenges, however, we believe are outweighed by the perceived benefits algorithms can provide to the overall evaluation of the evidence. To some extent, as hinted by PCAST, ignoring the calls for algorithms and failing to implement them as a means of empirically substantiating subjective judgment could be inevitably consequential to the enduring validity and admissibility of forensic evidence [8]. However, blindly implementing without careful planning and preparation could disastrous. For that reason, we turn our attention to *how* algorithms can be implemented into forensic practice in a *responsible* and *practical* manner. A responsible implementation requires consideration of issues from a quality assurance perspective to ensure the appropriate foundation has been laid out to support the implementation. Oftentimes, focus is directed toward whether the candidate method has been “validated” or “fit for purpose.” In our view, this is too narrow of a focus and, without further context, too broad of a question (i.e., what is the intended purpose and what is considered “fit” for such purpose?). A proper foundation requires a formalized quality management system be in place to ensure conformance with requisite requirements related to: education, training, protocols, validation, verification, competency, and on-going monitoring schemes. Specific guidelines related to each of these elements are available in other sources and within the context of specific examples, such as algorithms for DNA mixture interpretation (e.g., see Refs. [[97], [98], [99]]). For purposes of this discussion, each of these key topics are discussed in a more generic sense below.

The first pillar for a responsible implementation is to ensure practitioners and other stakeholders have foundational *education* related to the principles and theory underpinning the algorithm and quantification of the forensic observations, such as probability, statistics, uncertainty, and logic and reasoning. This education is distinct from training on the application of a specific algorithm and should be applied broadly to both practitioners, criminal justice and legal stakeholders, and, if possible, the public at large. For practitioners, this education should enable them to understand and articulate the epistemic limits of the evidence to which the algorithm is being applied and inferences that can be formed during evaluation. For legal stakeholders, this education should enable judicial actors to understand how algorithms could be applied, how to evaluate the reliability of a given method (e.g., through key performance characteristics represented in validation and verification materials), and the extent to which the algorithms can and should inform their ultimate judicial determinations. For the public at large, this education should expose the public to the realities of forensic evidence interpretation and the role algorithms can play in that process so they have an understanding of the strengths and limitations of forensic evidence for which the algorithms are applied.

The second pillar for a responsible implementation is to ensure practitioners have proper *training* on the algorithm, including appropriate applications of the algorithm. Specifically, to the extent possible, practitioners should understand and explain how the algorithm works, such as what features are taken into account, how they are accounted for, and how the output is calculated and the extent to which outputs might change as inputs vary. Additionally, practitioners should understand the key performance characteristics of the algorithm and the contexts under which those were tested to ensure the data are representative of real-world applications and the circumstances for a given case. In situations where algorithms operate as black-boxes, practitioners may not have a complete understanding of the innerworkings of the algorithm, but should have, at a minimum, a conceptual understanding of the details outlined above in order to understand the applicability and strengths and limitations of the system.

The third pillar for a responsible implementation is to ensure written *protocols* are in place to ensure the algorithm is applied correctly, consistently, and appropriately to evidence in a given case. Protocols related to the standard operations of the algorithm, interpretation guidelines, reporting standards, technical review, and adjudication of conflicts between practitioners’ subjective assessments and algorithmic outputs, should be available and publicly accessible. Protocols should clearly articulate what is permissible for input into the algorithm, when the algorithm should be applied, and how the results should be interpreted and accounted for in an overall report. Limitations related to the application of the algorithm and interpretation of the results should also be available and publicly accessible.

The fourth pillar for a responsible implementation is to ensure the algorithm has been subject to an appropriate *validation* to demonstrate its key performance characteristics and “fit for purpose” in a given application. This is a broad term that applies to the foundational validation of the algorithm in terms of its conceptual design, software implementation, and representativeness of casework applications. Prior to validation, the purpose of the algorithm and how it is intended to be applied should be clearly defined to enable a determination of whether the key performance characteristics are acceptable for the intended purposes. The conceptual design of the system should address how the algorithm works, such as what features are taken into account, how they are accounted for, and how the output is calculated and the extent to which outputs might change as inputs vary. The software implementation relates to the accurate coding of the algorithm into a software code for execution. Validation of correct implementation can be done by testing the execution of the software under controlled conditions for which a specific output is expected given the inputs or by a review of the source-code. To enable this, both the software executable *and* the source-code should be made publicly accessible for independent review and testing, including the datasets that have been used in the validation effort. If the source-code is not able to be made publicly available, then it is even more critical that, at a minimum, the software executable is available. The key performance characteristics of the algorithm (e.g., sensitivity, specificity, repeatability, reproducibility) and the parameters for which the key performance characteristics are calculated (e.g., decision thresholds, etc.) should be derived through empirical testing of the algorithm using samples for which ground truth are known and which are representative in type, quality, and condition of those for which the algorithm will be applied in casework, as applicable (i.e., input samples should vary in type, quality, and condition to the extent that differences in these attributes are accounted for by the algorithm and will impact the output). If the algorithm requires training data (e.g., AI/ML systems), the samples used for testing should be distinct from those used during training. Uncertainty in the calculated performance characteristics should be accounted for and available in the validation documentation. Meuwly et al. provide a detailed guideline for approaching validation for evidence evaluation methods which we consider to be a reasonable framework for addressing these issues [100]. Although the focus is specific to those methods which produce a likelihood ratio, the concepts are applicable to the development and validation of many algorithmic methods designed to assist with evidence evaluation and forensic interpretation [100]. In particular, they address key questions such as “what to measure?” (i.e., performance characteristics), “how to measure?” (i.e., performance metrics), and “what should be observed or deemed satisfactory?” (i.e., validation criterion) [100].

The fifth pillar for a responsible implementation is to ensure the algorithm has been subject to an appropriate *verification* to demonstrate the validity of the system when applied by specific end-users in accordance with a specific set of protocols and in a specific operating environment. Verification (often referred to as internal validation) is not intended to be a repeat of the foundational validation as described above. Rather, it is intended to demonstrate that the system is robust to applications in a specific context (e.g., people, training, protocols) and the key performance characteristics derived during validation are applicable to the conditions and circumstances for which it is applied operationally.

The sixth pillar for a responsible implementation is to ensure the individuals using the algorithm have demonstrated *competency* related to the algorithm, its application, and interpretation of results. Collectively, the pillars of education and training form the foundation for practitioners’ knowledge related to the algorithm. Competency testing provides a means of evaluating whether an individual has acquired and demonstrated the requisite knowledge related to the algorithm as well as the ability to apply the algorithm operationally in accordance with applicable protocols and within the limits of its validation. Competency testing should be measured against a minimum standard for acceptable performance and be conducted prior to operational deployment of an algorithm by a specific individual.

The seventh pillar for a responsible implementation is to ensure the algorithm and its application operationally is subject to on-going *monitoring* through proficiency testing and audits of casework applications. The on-going monitoring should account for both the algorithm and its application by practitioners. This monitoring should include (i) routine verification of the software implementation of the algorithm to ensure software or hardware changes do not impact its execution, (ii) the relevance and appropriateness of protocols governing the application of the algorithm, and (iii) practitioners’ knowledge and abilities to correctly apply the algorithm. This monitoring should be robust enough to detect vulnerabilities or problems with the algorithm or its application necessitating preventive or corrective action. Finally, the quality assurance program should be agile enough to improve when preventive or corrective actions are warranted.

In addition to ensuring the necessary foundations are in place from a quality assurance perspective to enable the implementation of algorithms, the next task is to identify a *practical* implementation scheme addressing how the algorithm will be deployed operationally. This should include where in the examination scheme the algorithm will be implemented and the manner in which the outcome of the evaluation will be reported to criminal justice stakeholders. As indicated earlier, the deployment of an algorithm may not necessarily need to be a binary choice of “all or nothing” (*either* the human *or* the algorithm). Instead, implementation can take many different forms with varying degrees to which the algorithm impacts the overall outcome of the evaluation. Decisions related to how the algorithm will be deployed will depend on the intended purpose the algorithm (i.e., is the output of the algorithm intended as the *sole-basis* for the evidential information or is it intended to be used as a *supplemental basis* for the information?), the performance characteristics of the algorithm (i.e., is the algorithm appropriate or “fit” for the intended purpose), and consideration of the tradeoff between the potential benefits of the algorithm and perceived risks for a given deployment scheme. Consideration of these issues will be discipline and context dependent. For purposes of exploring this issue further, we will do so against the backdrop of friction ridge examination. We recognize, however, that this discussion is likely applicable across several other pattern evidence domains.

For context, friction ridge examination is traditionally carried out by human experts and interpretations are based solely on their subjective judgment. Empirical measurements are often not taken and detailed standards for conclusions are non-existent leaving the ultimate determination up to the opinion of the expert. Consequently, assessments made during friction ridge examinations are susceptible to variation from one analyst to another (inter-analyst) as well as by the same analyst from one examination to another (intra-analyst). When considering borderline impressions with marginal quality, these variations might result in differences in the overall conclusion. In the broad spectrum, however, while the lack of empirical measurements and standards do not necessarily mean the practice as a whole is unreliable or fraught with error, it does raise questions as to how reliable the evidence is for the case at hand. Thus, there is a need for the friction ridge community to move towards integrating tools to quantitatively assess the quality and strength of friction ridge impression evidence to enable standardization and provide empirical substantiation to analysts’ claims. As it relates to the implementation of algorithms, fortunately, this can be accomplished in several different ways and does not require algorithms to completely supplant the role of the expert. Precisely *how* algorithms should be integrated into standard operating procedures and the implications to practice, however, is an open question. Dror and Mnookin [101] briefly touch on this in the context of Automated Fingerprint Identification Systems (AFIS) databases that have become ubiquitous tools for practitioners over the last several decades to enable more efficient searching, storage, and retrieval of friction ridge impressions and known exemplars. As it relates to algorithms for decision making, however, we propose there are three key issues that ultimately govern how algorithms can be applied in practice: (i) whether algorithms are applied before or after the expert has conducted a traditional examination and formed a subjective opinion, (ii) the extent to which the reported result was dependent upon the output of the algorithm, and (iii) the manner in which conflicting outcomes between the expert’s judgement and the algorithm’s output are resolved. We recognize that an additional point of debate is how forensic conclusions and statistical information (e.g. the output of an algorithm) should be articulated to fact-finders and other criminal justice stakeholders (e.g., see Refs. [[102], [103], [104]]). While we view that as important, we consider it beyond the scope of the current issue. This is because irrespective of how algorithms are implemented into practice, the manner in which results are articulated to fact-finders can be quantitative or qualitative, each having benefits and limitations, and deserving of a separate discussion.

Taking into account the factors outlined above, we propose to approach the issue of algorithm implementation, and the different ways algorithms can be implemented, similar to how the automotive industry approached the issue of autonomous driving: describing a formal taxonomy of six different levels of automation (i.e., algorithm influence) ranging from Level 0 (no algorithm influence) to Level 5 (complete algorithm influence). Each level represents a gradual transition from human to machine as the basis for forensic conclusions. For pattern evidence disciplines (including friction ridge examination), we propose the six different levels are: Level 0 (No Algorithm), Level 1 (Algorithm Assistance), Level 2 (Algorithm Quality Control), Level 3 (Algorithm Informed Evaluation), Level 4 (Algorithm Dominated Evaluation), Level 5 (Algorithm only). In levels 0 through 2, the human serves as the predominant basis for the evaluation and conclusion with increasing influence of the algorithm as a supplemental factor for quality control (used *after* the expert opinion has been formed). In Levels 3 through 5, the algorithm serves as the predominant basis for the evaluation and conclusion with decreasing influence from the human. The relationship between human and algorithm as well as the basis for conflict resolution and reported conclusions for each level is summarized in Table 1 and described in the discussion that follows.

**Table 1:** Levels of algorithm implementation describing the relationship between human and algorithm as well as the basis for conflict resolution and reported conclusions for each level.

Level	Name	Narrative Definition	Human Role	Algorithm Role	Conflict Resolution	Basis for Conclusion
0	No algorithm	The human is responsible for forming an expert opinion based on subjective observations without any use of the algorithm.	Evaluation	N/A	N/A	Expert Opinion
1	Algorithm Assistance	The human is responsible for forming an expert opinion based on subjective observations. The algorithm *may* be used *after* an initial opinion has been formed. The algorithm serves as an optional assistance tool supplemental to the expert opinion that may be used at the discretion of the examiner.	Evaluation	Supplemental Assistance (optional)	Expert Discretion	Expert Opinion
2	Algorithm Quality Control	The human is responsible for forming an expert opinion based on subjective observations. The algorithm *shall* be used *after* the opinion has been formed. The algorithm serves as a required quality control supplemental to the expert opinion to ensure the evidence conforms to specified criteria supporting a conclusion.	Evaluation	Supplemental Quality Control	Standard Operating Procedures	Expert Opinion (Algorithm Supported)
3	Algorithm Informed Evaluation	The human is responsible for forming an expert opinion based on the output of the algorithm. The algorithm *shall* be used *before* the opinion has been formed. The algorithm serves as an integrated factor informing the opinion.	Human-Algorithm Integrated Evaluation	Human-Algorithm Integrated Evaluation	Standard Operating Procedures	Algorithm Output (Human Supported)
4	Algorithm Dominated Evaluation	The algorithm is used as the basis for the conclusion. The human serves in an oversight capacity to ensure the algorithm is applied appropriately.	Procedural Oversight	Evaluation	Standard Operating Procedures	Algorithm Output
5	Algorithm Only	The algorithm is used as the basis for the conclusion without any human evaluation or oversight.	N/A	Evaluation	N/A	Algorithm Output

Level 0 characterizes traditional practices in the majority of forensic science disciplines. Besides the use of automated tools, such as AFIS to augment searching, storage, and retrieval tasks, algorithms do not have any substantive role in the evaluation of the evidence. The conclusion is based solely on the subjective opinion of the expert. This level is deeply rooted in tradition and represents the vast majority of forensic practitioners today (with the exception of DNA). Although practitioners are most comfortable with this approach, it has been the focus of increasing criticism from scientific and legal actors for the lack of statistical support.

Level 1 represents the lowest level of algorithm implementation. The human relies on traditional practices for the evaluation of the evidence and is responsible for forming an opinion independent of the algorithm. The expert may then use the algorithm *after* the initial opinion has been formed as an optional quality control. The human is considered the ultimate authority on the overall conclusion and is given complete discretion when to run the algorithm and how the output of the algorithm is considered. Conflicts between the expert’s judgment and the algorithm output are not required to be formally adjudicated by standard operating procedures. At most, conflicts between the expert’s judgment and the algorithm output may cause the expert to seek a second opinion through formal procedures of consultation or verification; however, the output of the algorithm is not a formal component of the examination scheme and therefore the results are not part of the reported conclusion. Since the algorithm is applied after the expert has formed their opinion and the algorithm is not part of the basis for interpretation or the reported conclusion, courts are unlikely to be concerned with the algorithm. This level may be appropriate when practitioners have not had any prior experience with algorithms. The key benefit of this level is that it provides flexibility for when and how the algorithm is used and a means for practitioners to slowly gain comfort with the algorithm and trust in the output. The key limitation to this level is that there is no formal mechanism to ensure experts are not improperly discounting the algorithm when it might conflict with their subjective assessment.

Level 2 represents a level of implementation in which the algorithm is used as a quality control for the ultimate conclusion reported. The human relies on traditional practices for the evaluation of the evidence and is responsible for forming an opinion independent of the algorithm. The expert then uses the algorithm *after* the opinion has been formed as a *required* quality control supplemental to the expert opinion to ensure the evidence conforms to specified criteria supporting a conclusion. In this scenario, the ultimate authority on the reported conclusion is governed by the standard operating procedures. In order for a particular conclusion to be warranted, the expert opinion must be supported by the algorithm output and conforming to criteria specified by the standard operating procedures (e.g., minimum threshold for a quantitative algorithm output). If the expert opinion is not supported by the algorithm, then the conflict is formally adjudicated in accordance with the standard operating procedures. The protocols proposed by Montani et al. [105] to provide a reasonable framework for addressing conflicts between human and algorithm within standard operating procedures. Although the algorithm has a material impact on the overall conclusion reported from a quality control standpoint, since it was applied after the expert has formed their opinion and therefore is not part of the basis for interpretation, courts are less likely to be concerned with the algorithm. The admissibility of the algorithm may be challenged; however, even if the algorithm was found to be inadmissible (e.g., novel algorithms that are not widely adopted and therefore are not yet “generally accepted”), it is unlikely to materially affect the admissibility of the evidence overall since the ultimate conclusion reported is still based on the expert opinion. This level is appropriate when practitioners have gained some experience with the algorithms and have established reasonable trust in the output. The key benefit of this level is that the algorithm is implemented in a way that provides empirical support for the expert opinion, but does not alter traditional interpretation practices related to the expert opinion. The key limitation to this level is that the expert does not have the opportunity to leverage the output of the algorithm as a factor when evaluating the overall value of the evidence.

Level 3 represents a key transition point between human and algorithm. In Level 2, the algorithm was used supplemental to the expert opinion (*after* the expert formed the opinion). At this level, the algorithm is used *before* the opinion has been formed. In this scenario, the algorithm serves as factor informing the expert opinion; thus, the expert has the benefit of being able to incorporate the output of the algorithm along with their subjective judgment. The ultimate authority on the reported conclusion is governed by the standard operating procedures. In order for a particular conclusion to be warranted, the algorithm output must conform to criteria specified by the standard operating procedures and must be supported by the expert opinion. If the algorithm output is not supported by the expert opinion, then the conflict is formally adjudicated in accordance with the standard operating procedures (e.g., see Montani et al. [105]). Since the algorithm is applied before the expert has formed their opinion and therefore serves as a basis for interpretation, courts are more likely to be concerned with the algorithm at this level than in lower levels. The admissibility of the algorithm may be challenged since it was a factor taken into consideration when forming the expert opinion; however, similar to lower levels, if the algorithm were found to be inadmissible, it is less likely to materially affect the admissibility of the evidence overall since the algorithm output was one of many factors taken into account when forming the expert opinion. This level is appropriate when practitioners have gained considerable experience with the algorithm and have established trust in the output. The key benefit of this level is that the algorithm is implemented in a way enables the expert to leverage the output of the algorithm as a factor when evaluating the overall value of the evidence. The key limitation to this level is that the interpretation remains dependent on subjective elements from the expert.

Level 4 represents a level of implementation in which the algorithm is used as the basis for the ultimate conclusion reported. In this scenario, the human does not form an expert opinion; rather, the expert determines whether the circumstances of the evidence are appropriate for the application of the algorithm and ensures it is applied correctly and in accordance with standard operating procedures. The ultimate authority on the reported conclusion is governed by the standard operating procedures. Since the algorithm serves as the basis for the conclusion, courts are more likely to be concerned with the algorithm at this level than in lower levels. The admissibility of the algorithm may be challenged since it served as the basis for the reported conclusion. Experts will need to have in-depth knowledgeable about the algorithm and be able to be responsive to questions and challenges to the weight of the evidence during testimony. At this level, if the algorithm were found to be inadmissible, it is likely to materially affect the admissibility of the evidence overall since the algorithm output was the basis for the ultimate conclusion. This level is appropriate when the technology is capable of this type of autonomy and practitioners have gained expert knowledge and experience with the algorithm and have established trust in the output. The key benefit of this level is that the algorithm is implemented in a way enables the expert to oversee the process and ensure appropriate application of the algorithm while the reported results are based on the algorithm output rendering them less susceptible to variations caused by human interpretation. The key limitation to this level is that courts may be *less* receptive to algorithms that operate or manifest as a “black-box” and are difficult to explain how the algorithm generated a particular result.

Level 5 represents the highest level of algorithm implementation for which the algorithm operates in a “lights-out” mode without any human involvement or oversight. In this scenario, the algorithm is fully autonomous and reported results are automatically generated by the machine. The admissibility and weight of the results of the algorithm may be challenged since it operates fully autonomously. At this level, if the algorithm were found to be inadmissible, it is almost certain to materially affect the admissibility of the evidence overall since the algorithm was the sole basis for the ultimate conclusion. This level is appropriate for high-performance algorithms and high-throughput operations where this level of automation is necessary and stakeholders have been informed, understood and accepted the benefits and risks associated with such deployment. The key benefit of this level is that the reported results are based entirely on the algorithm output and completely objective. The key limitation to this level is that practitioners are completely supplanted by the algorithm and without an expert able to testify to the application and overall process, courts are unlikely to be receptive to algorithms as a basis for substantive evidence that lack transparency and explainability to how the algorithm generated a particular result.

The levels of algorithm implementation summarized in Table 1 and described above illustrate different ways in which algorithms can be implemented—each with different implications to practice. On the one hand, from a scientific perspective, practitioners should swiftly move toward implementing algorithms at higher levels such that the algorithms provide the predominant basis for conclusions. Doing so would promote improved objectivity and consistency in the reported results. On the other hand, practitioners and courts are unlikely to be receptive to such a swift transition and become almost entirely dependent on algorithms without having the opportunity to gain comfort with the systems and establish trust in the outcome. Further, at higher levels of implementation, practitioners are likely to be expected to have a greater depth and breadth of knowledge about the algorithm and be responsive to questions and challenges during testimony. This may be concerning for practitioners that have traditionally required very little to no need for formal education in algorithms and statistical principles. We propose that the optimal approach is for practitioners to identify a target level of implementation that is practical given the current state of available technology and consideration of the tradeoff between the potential benefits and perceived risks for a given deployment scheme, then establish a plan for implementation that begins with Level 1 as a pilot phase and progresses sequentially through the various levels toward the target. Doing so will allow practitioners to gradually gain comfort with the systems, trust in the outcome, and time to increase their depth and breadth of knowledge that will be expected of them during testimony.

Using friction ridge examination as an example, given the current state of technology available for implementation, target levels of implementation might include Levels 2 or 3—either of which are achievable [37,106] and least impactful to traditional practices. Level 1 should be short-lived as a pilot phase and first step to gain initial comfort with the system and evaluate the performance of the algorithm when applied operationally. Level 4 is possible as a target given current technology, but likely unsettling to many practitioners and some stakeholders since available algorithms do not fully account for all the types of features and distinguishing characteristics practitioners are able to take into consideration during their subjective assessments. Aside from high-quality samples, such as known-to-known comparisons (i.e., “ten-prints”), Level 5 implementation is likely not practical given the current state of available technology for latent print impressions involving partial and degraded samples. In addition to the technology considerations yielding Levels 2 and 3 as optimal targets, they offer several other benefits that appear to balance the interests and needs of the various stakeholders. Some of these benefits include: (i) practitioners are more likely to adopt algorithms since they remain empowered to express their expert opinion, (ii) fact-finders would no longer be required to rely on testimony *ipse dixit* as the algorithm would provide a means of ensuring analysts’ opinions to be empirically supported, (iii) the resource burden on forensic laboratories that would be necessary for educating and training practitioners related to the underpinnings of the algorithm and statistical concepts is minimal compared to what would be necessary to ensure a depth of knowledge necessary to support Level 4 or 5 implementation, (iv) courts are less likely to be faced with resource-intensive admissibility challenges or concerns of Constitutional infringements since the algorithms are merely supplemental to the evaluation of the evidence (versus the predominant basis thereof), and (v) the admissibility of the algorithm can be considered distinct from the admissibility of the expert opinion. As the technology advances in coming years and practitioners (and other criminal justice stakeholders) become more acclimated with the use of algorithms in forensic science, the expectation is that implementation schemes will continue to progress toward higher levels. Irrespective of the level of implementation, however, the expert will remain critical as a steward to the overall process and necessary for the admissibility of the evidence under the Sixth Amendment [93].

## 2 Conclusion

The implementation of algorithms (e.g., statistical methods) in forensic science is complicated. Although scientific and legal scholars have raised concern that many forensic conclusions lack empirical support and researchers have proposed various statistical or algorithmic approaches, the practitioner community has been reluctant to apply them operationally and their implications to litigation have yet to be fully demonstrated. Reactions from practitioners to statistical interventions have ranged from passive skepticism to outright opposition, often in favor of traditional experience and expertise as a sufficient basis for conclusions. In this paper, we explored *why* practitioners are generally in opposition to algorithms and *how* their concerns might be overcome. We accomplished this by considering issues concerning human—algorithm interactions in both real world domains and laboratory studies as well as issues concerning the litigation of algorithms in the American legal system. Ultimately, recognizing the need to heed the calls for algorithms is inevitable, we propose *how*, not *if*, algorithms could be implemented into operational practice that is both *responsible* and *practical*, such that forensic practitioners and other legal and scientific stakeholders are likely to accept.

Following our exploration of the different issues, we made several observations that enabled us to characterize key challenges to implementation. On the topic of human—algorithm interactions, we found that people tend to exhibit a general aversion to algorithms and prefer to rely on their own judgment—often despite knowledge that their own judgment is typically inferior to that of algorithms. This phenomenon is exacerbated when people possess domain expertise, are faced with high-stakes decisions, and are presented with an algorithm that is susceptible to err. Indeed, forensic science has the conditions for which algorithm aversion is most pronounced. From both anecdotal observations of human—algorithm interactions in different domains and recent research, we found that people tend to be more receptive to algorithms if they are integrated as a factor that *supplements* as opposed to *supplants* human decision making and the human retains some amount of influence on the ultimate outcome. On the topic of litigating algorithms in the American legal system, we found that this can be particularly challenging given the “black-box” nature of many algorithms and, in some cases, the countervailing legal protections against disclosure of the actual algorithm and source-code. The opacity of algorithms will often trigger admissibility challenges as well as raise concerns over infringements to Defendants’ Constitutional rights provided by the Fifth and Sixth Amendments.

In our view, despite these issues, algorithms will ultimately be inevitable to ensure the enduring validity and admissibility of forensic evidence for decades to come. In recent years many forensic science disciplines have been put on notice by formal bodies expressing concern from scientific and legal perspectives that expert opinions need to be empirically supported with statistical data (e.g., see Refs. [3,[7], [8], [9]]). An abrupt shift requiring immediate implementation of statistical and algorithmic methods as a condition for admissibility would be impractical and unrealistic; however, we believe it will only be a matter of time until patience wears and courts limit deference to experts and accept opinions *ipse dixit*. Recognizing the inevitable need for algorithms in forensic science and taking into consideration the issues concerning human—algorithm interaction and litigation of algorithms, we propose a strategy for approaching the implementation of algorithms in a *responsible* and *practical* manner by: (i) outlining the foundations that need to be in place from a quality assurance perspective before algorithms should be implemented, such as education, training, protocols, validation, verification, competency, and on-going monitoring schemes; and (ii) proposing a formal taxonomy of six different levels of algorithm implementation ranging from Level 0 (no algorithm influence) to Level 5 (complete algorithm influence) describing various ways in which algorithms can be implemented, similar to how the automotive industry approached the issue of autonomous driving. Each level represents a gradual transition from human to machine as the basis for forensic conclusions and include: Level 0 (No Algorithm), Level 1 (Algorithm Assistance), Level 2 (Algorithm Quality Control), Level 3 (Algorithm Informed Evaluation), Level 4 (Algorithm Dominated Evaluation), Level 5 (Algorithm only). In levels 0 through 2, the human serves as the predominant basis for the evaluation and conclusion with increasing influence of the algorithm as a supplemental factor for quality control (used *after* the expert opinion has been formed). In Levels 3 through 5, the algorithm serves as the predominant basis for the evaluation and conclusion with decreasing influence from the human. We propose the optimal approach is for practitioners to identify a target level of implementation that is practical given the current state of available technology and consideration of the tradeoff between the potential benefits and perceived risks for a given deployment scheme, then establish a plan for implementation that begins with Level 1 as a pilot phase and progresses sequentially through the various levels toward the target. Proceeding in this fashion will increase the likelihood for adoption across all stakeholders and lead to an overall stronger foundation and improvement to the quality and consistency of forensic science.

## Declaration of competing interest

The authors declare that they have no known competing financial interests or personal relationships that could have appeared to influence the work reported in this paper.

## Footnotes

1 In other words, to achieve greater performance overall through algorithms, we may need to tolerate reduced performance through human intervention in order to increase the tendency for people to rely on them.
